# Peer Review of “All You Need Is Context: Clinician Evaluations of Various Iterations of a Large Language Model–Based First Aid Decision Support Tool in Ghana (Preprint)”

**DOI:** 10.2196/65727

**Published:** 2024-09-18

**Authors:** Yixuan Gao, Toba Olatoye, Randa Salah Gomaa Mahmoud

**Affiliations:** 1 Department of Computer Science, Cornell University, 616 Thurston Ave, Ithaca, NY, United States, 1 607-254-4636; 2 University of Ilorin, Ilorin, Nigeria; 3 Zagazig University, Zagazig, Egypt

**Keywords:** medical informatics, clinical decision support tools, AI in health care, large language models, emergency medical services, clinical evaluation, medical emergencies, resource-constrained settings

*This is a peer-review report submitted for the preprint “All You Need Is Context: Clinician Evaluations of Various Iterations of a Large Language Model–Based First Aid Decision Support Tool in Ghana.”*

This review is the result of a virtual collaborative live review discussion organized and hosted by PREreview and JMIR Publications on June 20, 2024. The discussion was joined by 15 people: 2 facilitators, 2 members of the JMIR Publications team, 2 authors, and 9 live review participants, including 3 who agreed to be named, Aswathi Surendran, Khushboo Thaker, Arya Rahgozar, and Emmanuel Adamolekun, but did not contribute to the final composition of this review. The authors of this review have dedicated additional asynchronous time over the course of 2 weeks to help compose this final report using the notes from the live review. We thank all participants who contributed to the discussion and made it possible for us to provide feedback on this preprint.

## Summary

This study [1] investigates the performance and application of large language models (LLMs) as support tools for making clinical decisions during medical emergencies in the resource-constrained settings of low- and middle-income countries (LMIC) such as Ghana. The research’s aim is to provide a premise for future research and development of LLM-based clinical decision support tools by assessing the suitability and effectiveness of five selected generalized LLMs using context-specific prompts. A total of 13 medical experts with an average of 3 years of experience working in an environment of limited resources evaluated the outputs of these models quantitatively by using mean ranking scores and qualitatively using thematic analysis.

The authors used off-the-shelf pretrained LLMs (GPT-4 Turbo, Gemini 1.5 Pro, and Claude Sonnet) with prompt engineering and retrieval augmented generation (RAG) techniques to develop five iterations of a decision support tool. A total of 50 responses were generated and evaluated. Machine evaluations were also performed and compared with theirs, using conventional machine learning metrics like bilingual evaluation understudy and Recall-Oriented Understudy for Gisting Evaluation.

Their findings showed that Gemini 1.5 Pro+ prompt engineering outperformed the other LLMs used in their research, while the adjustments of other LLMs using suitable parameters improved their overall performance. This may imply that LLM-based first aid assistants could provide useful instructions for the management and treatment of medical conditions, especially in resource-constrained settings. The practitioners were generally satisfied with the diagnoses and instructions from these LLMs, demonstrating their potential and importance in managing medical emergencies. Future research should involve larger datasets, additional metrics, and more detailed evaluations to refine and enhance the use of LLMs in real-world medical emergencies.

The discussion from participants of this live review is summarized below.

## List of Major Concerns and Feedback

### Statistical Significance of Differences in Mean Ranking Scores

- Concern: The paper does not assess if the difference in mean ranking scores with a change in RAG approach (result in Table 2) is statistically significant.
- Feedback: Perform statistical tests such as *t* tests or Kruskal-Wallis test by ranks to determine if the differences in mean ranking scores are statistically significant. This will add robustness to the findings.

### Incomplete Figures

- Concern: The Figure 2 image is incomplete, with the right side cut off, and the Figure 1 legend is incomplete. In Figure 3, the data is not clear to assess the correlation.
- Feedback: Revise the figures to ensure they are complete and clearly labeled. This will improve the clarity and comprehensibility of the visual data.

### Availability of Google Form Reference

- Concern: The Google form (reference 15) is not available.
- Feedback: Ensure the Google form is accessible in the supplementary files. This is crucial for transparency and reproducibility.

## List of Minor Concerns and Feedback

- It would be helpful for the reader to see the aim of the work, the main results, and the conclusion mentioned in the abstract.
- Participants were a bit confused about reference 1 in the Authors section and wondered if that was the most appropriate place to cite the project involved with this study.
- It is unclear if Claude 3.5 Sonnet or Claude 3 Opus was used. Please clarify.
- It is unclear what is being referred to with “Low-and Low-Middle-Income countries (LMICs).” Is it low-income countries or “Lower Middle Income Countries (LMICs),” forms more commonly used as defined by the World Bank [2]?
- In section E of the Methodology, it would be helpful to mention the total number of clinicians involved in the study. In section G, the text says “The first group of 30 responses were evaluated by all 13 physicians. The second group of 20 responses was evaluated by 8 of the physicians.” It would be helpful to know why and how these 8 were selected out of the total 13.
- In section F of the Methodology section, the text presents a quote by one of the clinicians involved. It would be helpful to understand why this quote is presented in the text.
- It would be helpful to have more information about the statistical tests used for the quantitative analysis and why.
- In the Results section, there seems to be inconsistency in the labeling style of tables: Roman numerals in the text versus Arabic numerals in the figure label. It would be helpful to choose one style and be consistent throughout the manuscript so that the reader can better follow the results.
- In the Results section, under the Qualitative Analysis section, the sentence “Table 3 shows the 8 codes and their descriptions”: Table 3 should be corrected to Table 4.
- Figure 1 is a bit hard to read and understand. A bigger font and an explanation of what is plotted in the figure legend would significantly enhance comprehension.
- In the second paragraph on page 6, the abbreviation EMS is first mentioned and it should be spelled out as the emergency medical services (EMS).
- It was expected that the RAG-based approach would have performed better than the approach solely based on LLM. It would be helpful if the authors discussed the results in the context of these expectations, highlighting potential limitations of the study.

## Abbreviations

EMS: emergency medical services
LLM: large language model
LMIC: low- and middle-income countries
RAG: retrieval augmented generation
